# Waves of Calcium Depletion in the Sarcoplasmic Reticulum of Vascular Smooth Muscle Cells: An Inside View of Spatiotemporal Ca^2+^ Regulation

**DOI:** 10.1371/journal.pone.0055333

**Published:** 2013-02-07

**Authors:** Mitra Esfandiarei, Nicola Fameli, Yohan Y. H. Choi, Arash Y. Tehrani, Jeremy G. Hoskins, Cornelis van Breemen

**Affiliations:** Child & Family Research Institute, Department of Anaesthesiology, Pharmacology, and Therapeutics, University of British Columbia, Vancouver, British Columbia, Canada; University of Debrecen, Hungary

## Abstract

Agonist-stimulated smooth muscle Ca^2+^ waves regulate blood vessel tone and vasomotion. Previous studies employing cytoplasmic Ca^2+^ indicators revealed that these Ca^2+^ waves were stimulated by a combination of inositol 1,4,5-trisphosphate- and Ca^2+^-induced Ca^2+^ release from the endo/sarcoplasmic reticulum. Herein, we present the first report of endothelin-1 stimulated waves of Ca^2+^ depletion from the sarcoplasmic reticulum of vascular smooth muscle cells using a calsequestrin-targeted Ca^2+^ indicator. Our findings confirm that these waves are due to regenerative Ca^2+^-induced Ca^2+^ release by the receptors for inositol 1,4,5-trisphosphate. Our main new finding is a transient elevation in SR luminal Ca^2+^ concentration ([Ca^2+^]_SR_) both at the site of wave initiation, just before regenerative Ca^2+^ release commences, and at the advancing wave front, during propagation. This strongly suggests a role for [Ca^2+^]_SR_ in the activation of inositol 1,4,5-trisphosphate receptors during agonist-induced calcium waves. In addition, quantitative analysis of the gradual decrease in the velocity of the depletion wave, observed in the absence of external Ca^2+^, indicates continuity of the lumen of the sarcoplasmic reticulum network. Finally, our observation that the depletion wave was arrested by the nuclear envelope may have implications for selective Ca^2+^ signalling.

## Introduction

In vascular smooth muscle cells (VSMCs), fluctuations in cytoplasmic Ca^2+^ concentration ([Ca^2+^]_i_) selectively control multiple functions, including contraction-relaxation, energy metabolism, proliferation, migration and apoptosis, in health and disease [1], [2], [3], [4], [5]. It is generally accepted that functional selectivity of Ca^2+^ signals is encoded in their spatial and temporal characteristics [6], [7], [8]. In this context, we support the view that the ubiquitous asynchronous Ca^2+^ waves in VSM are optimally suited to couple agonist-mediated stimulation to vasoconstriction in healthy blood vessels, while avoiding recruitment of stress related functions [2], [3], [9], [10]. Neylon and coworkers were the first to report that receptor stimulation of cultured human VSM did not simply elevate [Ca^2+^]_i_ to induce activation, but in fact caused a wave of elevated [Ca^2+^]_i_ to travel across the cell from an initiation site [11]. They further proposed a mechanism involving stimulation of inositol 1,4,5-trisphosphate receptors (IP_3_R) and Ca^2+^ induced Ca^2+^ release (CICR) [11]. In 1994, Iino *et al.* made the next advance by recording [Ca^2+^]_i_ in the intact rat tail artery smooth muscle [4]. Iino had earlier discovered that IP_3_Rs were sensitive to Ca^2+^, such that IP_3_ required Ca^2+^ as a co-activator, and that the elevation of [IP_3_]_i_ facilitates CICR at IP_3_Rs [12]. Sympathetic nerve stimulation initiated asynchronous repetitive waves of [Ca^2+^]_i_ elevation, traveling in both directions along the length of the spirally arranged smooth muscle fibres. The unexpected aspect of this discovery was that the tonic increases in both the bulk smooth muscle [Ca^2+^]_i_ and force were based on wave-like [Ca^2+^]_i_ oscillations in individual VSMCs. Because of the asynchronous nature of these cellular Ca^2+^ waves, summation over thousands of cells in the vascular media results in maintained average [Ca^2+^]_i_ elevation and vascular tone. Nevertheless, the repetitive Ca^2+^ waves would still confer the advantage of added informational content due to frequency encoding and prevention of potential harm due to prolonged elevated [Ca^2+^]_i_.

Although a great deal of valuable information has subsequently accumulated on the mechanism of Ca^2+^ waves, previous studies were limited, to some degree, by the fact that they reported changes in cytoplasmic Ca^2+^, which represent the result of Ca^2+^ release from the sarcoplasmic reticulum (SR) rather than the process itself. In this study, in order to obtain more direct insight into regenerative Ca^2+^ release in VSMCs, we have used a specific Ca^2+^ indicator targeted to calsequestrin in the SR lumen that we refer to as D1SR. The VSMCs used in this study constituted a primary culture, which remained highly contractile during the study. Here, we report a wave of Ca^2+^ depletion from the SR itself, which in this case is activated by applying the vascular autocoid endothelin-1 (ET-1). The observed agonist-induced SR Ca^2+^ depletion wave provides valuable new information about the role of SR luminal Ca^2+^ concentration ([Ca^2+^]_SR_) in the initiation and propagation of Ca^2+^ waves, which was not attainable by recording fluctuations in [Ca^2+^]_i_.

## Materials and Methods

### Buffers & Reagents

HEPES-PSS containing (in mmol·L^−1^) NaCl 140, glucose 10, KCl 5, HEPES 5, CaCl_2_ 1.5 and MgCl_2_ 1 (pH 7.4) was used for all calcium measurements and confocal microscopy. The nominal zero-Ca^2+^ PSS was prepared in the same way as normal PSS without the addition of calcium. Endothelin-1 (ET-1) was obtained from Sigma-Aldrich (ON, Canada). Thapsigargin (Tg), a cell permeable inhibitor of sarcoplasmic reticular Ca^2+^-ATPase (SERCA), and xestospongin C (Xes-C), a potent membrane-permeable blocker of IP_3_-mediated Ca^2+^ release, were purchased from EMD Chemicals (NJ, USA). Sodium orthovanadate, an inhibitor of secretory pathway Ca^2+^ ATPase (SPCA) pumps, was purchased from Sigma-Aldrich. Stock solutions of ET-1 and Tg were prepared in dimethyl sulfoxide (DMSO). For all experiments, vehicle-treated (0 µM) groups were incubated with equal volume of DMSO, respectively (the maximum volume of solvents used with the highest concentration of drugs). Further dilutions of reagents were made in zero-Ca^2+^ PSS buffer. The calcium dye Fluo-4 AM, ER-Tracker, Mito-Tracker Green FM, BODIPY® TR-X thapsigargin, and CellLight™ Golgi-GFP BacMam 2.0 were all purchased from Invitrogen (ON, Canada).

### Cell Culture

Rat aortic SMCs were originated from the laboratories of Drs. Urs Ruegg and Nicolas Demaurex (University of Geneva, Geneva, Switzerland), and prepared from aorta of male Wistar Kyoto rats (200–300 g) as previously described [13]. Cells between passage 9 to 13 were cultured in Matrigel-coated (BD Sciences; ON, Canada) 35 mm glass bottom culture dishes (MatTek Co., MA, USA), and grown in Dulbecco's modified Eagle's medium supplemented with 10% heat-inactivated newborn calf serum, Penicillin G (100 µg.mL^−1^), and streptomycin (100 µg.mL^−1^) (Invitrogen, ON, Canada) at 37°C, in a humidified incubator in 5% CO_2_. See following sections for a detailed description of the transfection protocol and buffer/reagents used in the study.

### Transient Transfection with D1SR Constructs

D1SR indicator, a modified variant of the D1ER cameleon [14], [15], [16], is kindly provided by Dr. Wayne Chen (University of Alberta, Canada). In the D1SR indicator, the original calreticulin signal sequence in D1ER has been replaced by the mutant calsequestrin sequence with a reduced binding ability to calcium (to eliminate the competition with endogenous calsequestrin in binding to calcium ion within SR). The D1SR construct consists of a truncated enhanced CFP and YFP that are joined by a linker containing modified calmodulin (CaM) and M13 (the 26-residue CaM-binding peptide of myosin light-chain kinase) sequences. The CaM-M13 modifications prevent M13 from binding endogenous calmodulin. SR retention is achieved by calcequestrin sequences on the 5′ end of CFP. Following binding to Ca^2+^, conformational changes in the CaM-M13 domain increase the fluorescence resonance energy transfer (FRET) between the flanking CFP and YFP yielding a Ca^2+^ response. SMCs were transfected with adenoviral D1SR constructs at a multiplicity of infection of 100 (MOI = 100). Following overnight incubation at 37°C, cells were replenished with fresh medium. Fluorescence microscopy was used to assess transfection efficiency and cellular morphology at 48 h post transfection. For all experiments, a transfection efficiency of 80–90% was achieved.

### SR Luminal Ca^2+^ Measurement

Ratiometric FRET images were acquired with a 63× oil-immersion objective (Leica DM16000 inverted microscope) and a cooled Hamamatsu 9100-02 electron multiplier CCD camera. Cells were excited at 440 nm, and 513 nm; and imaged using 488 nm/535 nm (for donor and FRET channels) and 535 nm (for acceptor channel) band-pass filters (three images/time point). The acceptor channel was simultaneously recorded to monitor photobleaching. We set the intensity of light at 15% transmitted light, and excitation exposure times at 150 ms with 10 s intervals.

### Cytoplasmic Ca2+ Measurement

During *in vitro* measurement of cytoplasmic Ca^2+^ signals, all parameters (laser intensity, gain, etc.) were maintained constant during the experiment. The cell culture was illuminated using an Argon-Krypton laser (488 nm) and a high-gain photomultiplier tube collected the emission (505–550 nm). The customized Hamamatsu 9100-02 electron multiplier CCD camera delivers 1000×1000 pixels, imaged to Shannon-Nyquist specifications for the 63× objectives, providing a larger field of view. The representative fluorescence traces shown reflect the averaged fluorescence signals from 15 regions of interest (ROIs) in each cell. The measured changes in Fluo-4 AM fluorescence level are proportional to the relative changes in cytoplasmic Ca^2+^ ([Ca^2+^]_i_). The confocal images were analyzed off-line with the Improvision Volocity software (Perkin-Elmer). Fluorescence traces were extracted from the movies and were normalized to initial fluorescence values.

### Image Analysis

All data used for Ca^2+^ traces were analyzed by Improvision Volocity software, using built-in regions-of-interest (ROI) function to select the areas of interest that is at least 30 pixels long in length and width. For traces involving specific sites, shape and size of ROI were adjusted to avoid artifacts and saturated areas. For distance and velocity analysis, one ROI was selected per frame (time point) based on the movement of the Ca^2+^ wave. The resulting data were formatted on Microsoft Excel 2007, and analyzed by GraphPad Prism 5.0. Pseudo-colour visualization was performed by ImageJ, using customized lookup tables to assign colour for each pixel intensity values. Line scan was performed first by analyzing pixel intensity of a series of small ROIs (1–3 pixels in length per ROI) along a line using a customized Python script to output change in intensity over time along the line. The script was confirmed to output same values when analyzing same ROI on Volocity. The resulting values were then graphed using Gnuplot 4.4 with custom lookup table.

### Statistical Analysis

Data are presented as means ± SEM of at least four independent experiments. Significance was determined using Student’s *t*-test with two-tailed distribution using GraphPad Prism 5 (*P*≤0.05 was considered as statistically significant).

## Results

### Distribution of D1SR Ca^2+^ Indicator in Rat Aortic SMCs

**Figure 1:**
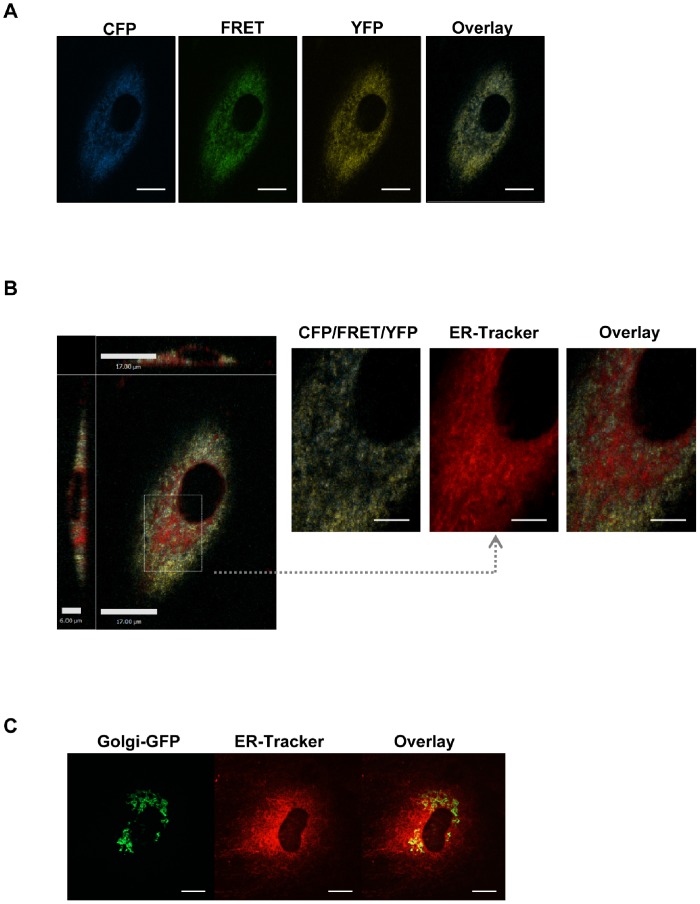
Distribution of D1SR Ca^2+^ indicator in rat aortic SMCs. **A)** Fluorescence images of D1SR indicator distribution using CFP (440/488 nm), FRET (440/535 nm), and YFP (514/535 nm) band-pass filters (scale bars, 10 µm). **B)** 2-D projection of a merged fluorescence image of D1SR using CFP (440 nm excitation/488 nm emission), FRET (440 nm excitation/535 nm emission), and YFP (514 nm excitation/535 nm emission) band-pass filters, and ER-Tracker™ Red (587 nm excitation/615 nm emission) are shown. The majority of area shows overlay of D1SR and SR lumen tracker. The zoomed-in images highlight areas of SR lumen negative for D1SR signal, but positive for ER-Tracker. In all experiments, such areas were excluded from data analyses (scale bars, 10 µm unless indicated otherwise). **C)** SMCs are loaded with Golgi-GFP CellLight® solution for 16 hours at 37°C prior to loading with ER-Tracker™ Red dye for 15 minutes. Fluorescence images of ER-Tracker™ Red (587 nm excitation/615 nm emission) and Golgi-GFP (488 nm excitation/520 nm emission) are shown (scale bars, 10 µm).

To monitor fluctuations in [Ca^2+^]_SR_, we transfected rat aortic SMCs with an adenoviral vector expressing the specific FRET-based SR Ca^2+^ indicator, D1SR. Figure 1A shows the individual and merged fluorescence images of three recorded channels (CFP: 440 nm/488 nm; FRET: 440 nm/535 nm; YFP: 513 nm/535 nm) for D1SR indicator in cultured VSMCs (see Movie S1). We also compared the distribution of D1SR with that of ER-Tracker, commonly used for localization of either ER or SR. As expected, D1SR co-localizes with ER-Tracker except for a region close to the nucleus that is negative for D1SR fluorescence, but positive for ER-Tracker (Fig. 1B). Specific staining with Golgi-Tracker indicated that the Golgi apparatus is typically located in this region (Fig. 1C).

### D1SR Calibration *in situ*

**Figure 2:**
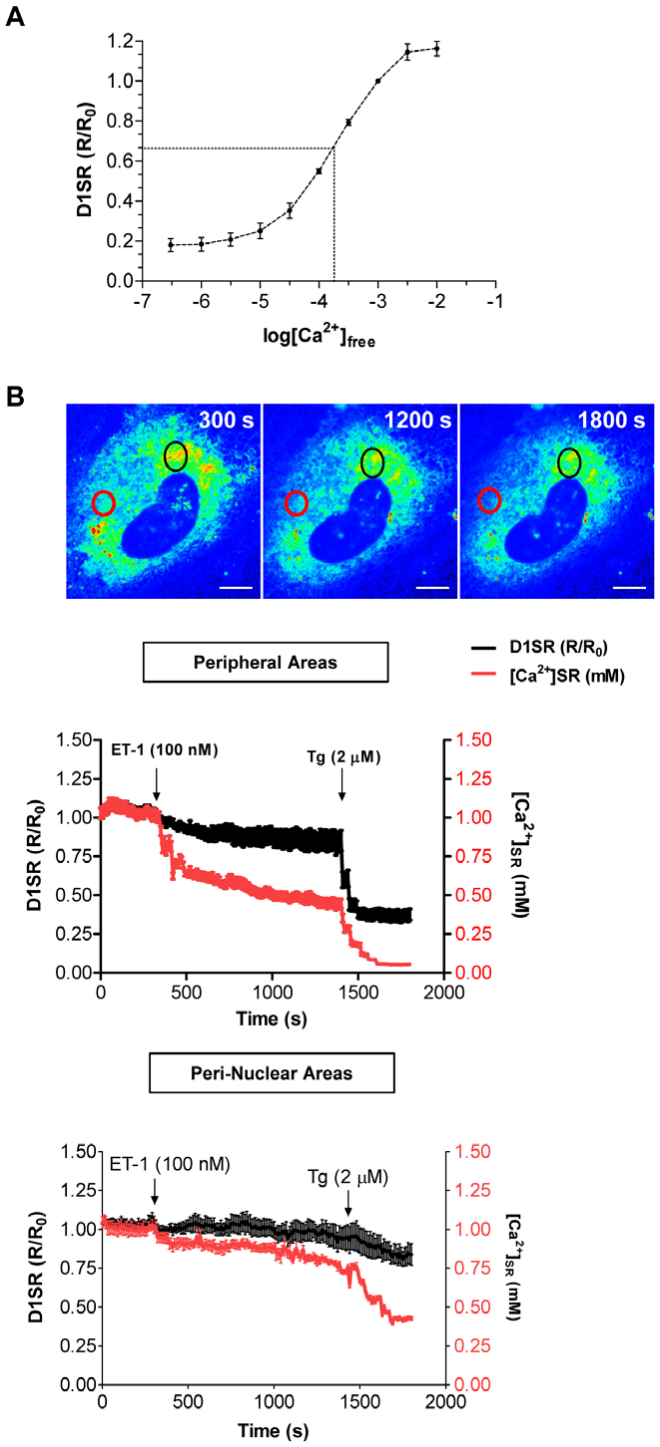
Calibration of D1SR *in situ* and effects of endothelin-1 and thapsigargin on SR Ca^2+^ signal. **A)** D1SR fluorescent ratio was calibrated *in situ* in semi-permeabilized SMCs (n = 12 cells from four independent culture plates, mean ± SEM) in intracellular solutions with 0.001 to 10 mM of free Ca^2+^, and fitted to an exponential equation. **B)** Selected snapshots from a time-lapse movie (10 s intervals) of rat SMCs transfected with D1SR indicator (scale bars, 5 µm), and averaged *R/R_0_* and corresponding [Ca^2+^]_SR_ values in peripheral (red circle) and peri-nuclear (black circle) areas in response to ET-1 (100 mM) and Tg (2 µM) treatments in the presence of extracellular Ca^2+^. As illustrated, ET-1 causes a slight change in *R/R_0_* values in the peri-nuclear area (ROIs = 20, n = 6 independent experiments, mean ± SEM, *P*<0.005).

The D1SR indicator was calibrated *in situ* in semi-permeablilized rat aortic SMCs in intracellular HEPES solution (135 mM KCl, 10 mM NaCl, 1 mM MgCl^2^, 20 mM HEPES, 20 mM sucrose, 0.01 mM digitonin, 0.01 mM ionomycin, 0.005 mM CCCP, PH 7.2) with 5 mM HEDTA as described before [16]. The intracellular solution was blended with the stock solution of 100 mM CaCl_2_ to prepare buffers with free Ca^2+^ concentrations ([Ca^2+^]_free_) ranging from 1 µM to 10 mM (Max Chelator v2.40, C. Patton, Standford University, USA, maxchelator.stanford.edu). D1SR fluorescent ratio (*R/R_0_*) was plotted against [Ca^2+^]_free_ in 12 cells from four independent cultures and an exponential fit was applied to the data to determine the apparent dissociation constant K_d_ (197±47 µM) and the Hill slope (*n = *0.87±0.19) (Fig. 2A). [Ca^2+^]_SR_ was calculated by calibrating normalized D1SR ratio against the standard calibration curve as described previously [16]. To calculate [Ca^2+^]_SR,_ normalized ratio values were fitted to:
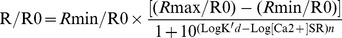
where *R_min_* and *R_max_* were obtained by measuring signal intensity in cells perfused with intracellular HEPES solution containing 5 mM HEDTA (Calcium free buffer) and 10 mM free calcium (15 mM CaHEDTA), respectively.

### Effects of Endothelin-1 and Thapsigargin on SR and Cytoplasmic Ca^2+^ Signals

The traces in Figure 2B illustrate the effects of receptor activation with ET-1 (100 nM) and SERCA inhibition with 2 µM thapsigargin (Tg) on [Ca^2+^]_SR_ in the peripheral (marked with red circle) and peri-nuclear (marked with black circle) areas of SMCs. In the cell periphery, ET-1 causes a drop in [Ca^2+^]_SR_ (from 1.08±0.12 mM to 450±50 µM), and subsequent SERCA inhibition results in an additional decline (to 55±11 µM). In the peri-nuclear areas, however, the ET-stimulated changes in [Ca^2+^]_SR_ are moderate (from 1.05±0.07 mM to 700±50 µM). Although a clear loss of [Ca^2+^]_SR_ is evident following Tg application, a considerable amount of D1SR signal remains in the peri-nuclear areas (427±23 µM), indicating the presence of organelles containing calsequestrin and Ca^2+^ that are not sensitive to SERCA blockade. A plausible explanation for this residual [Ca^2+^]_SR_ is that in these cells the SR is contiguous with membranous organelles or endosomes, which accumulate Ca^2+^ via the Tg-insensitive “secretory pathway Ca^2+^ ATPase” (SPCA). An alternative explanation is provided by a recent report from Ledeen’s group [17], which showed that NCX located in the inner membrane of the nuclear envelope is able to take up Ca^2+^ after SERCA blockade. Furthermore, since the nuclear envelope and the SR are confluent, this Ca^2+^, taken up by the NCX is capable of diffusing into the peri-nuclear SR.

**Figure 3:**
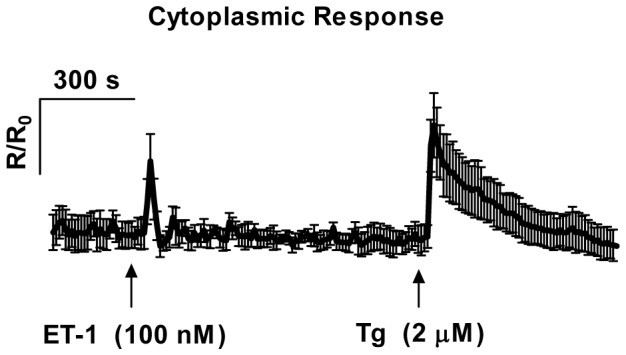
Effects of endothelin-1 and thapsigargin on cytoplasmic Ca^2+^ signal. Average trace of cytoplasmic Ca^2+^ signals recorded (10 s intervals) in rat SMCs loaded with Ca^2+^ indicator Fluo-4 AM. ET-1 (100 nM) initially causes a spike in [Ca^2+^]_i_, and subsequent inhibition of SERCA with 2 µM Tg elicits another large [Ca^2+^]_i_ transient due to depletion of SR luminal Ca^2+^ (ROI = 15, n = 4 independent experiments, mean ± SEM, *P*<0.005).

In order to compare the changes in [Ca^2+^]_SR_ with fluctuations in [Ca^2+^]_i_, we used the cytoplasmic Ca^2+^ indicator fluo-4 AM in the same VSMC preparation (Fig. 3). In these cells, ET-1 (100 nM) appears to be a weak agonist eliciting only a transient increase in [Ca^2+^]_i_ as opposed to uridine*-*5′-triphosphate (UTP), which stimulates a large maintained increase in [Ca^2+^]_i_. Subsequent inhibition of SERCA with Tg (2 µM) elicits another large [Ca^2+^]_i_ transient due to depletion of SR luminal Ca^2+^. Therefore, at least in the case of ET-1, it is clear that the luminal SR Ca^2+^ indicator shows dynamics, which are different from those observed with the cytoplasmic Ca^2+^ sensitive dye, fluo-4 AM.

### ET-1-induced Regenerative SR Ca^2+^ Depletion Waves in the Absence of Extracellular Ca^2+^

**Figure 4:**
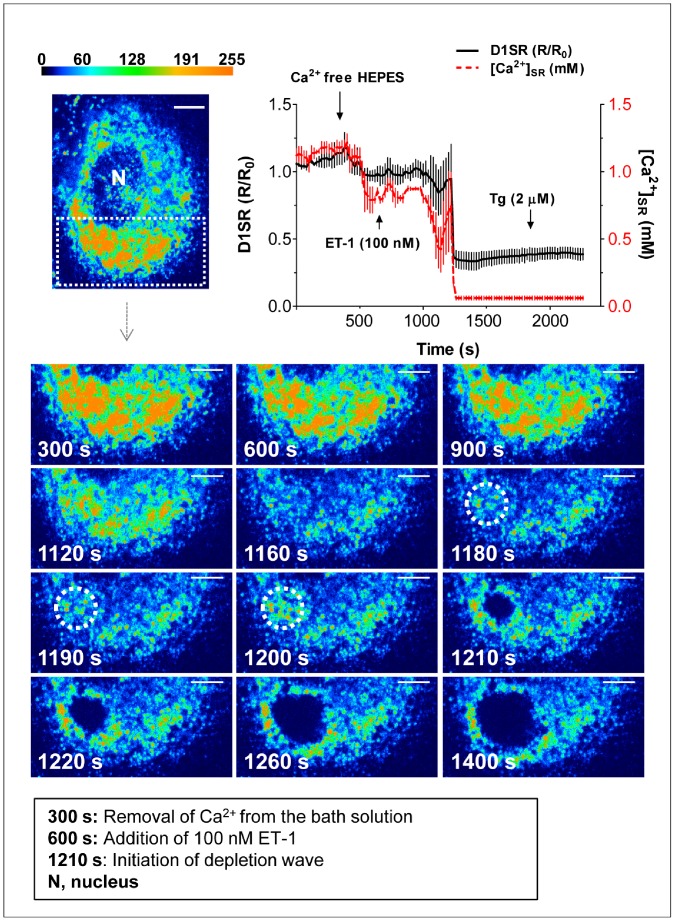
Progression of ET-1-induced regenerative Ca^2+^ depletion wave in the absence of extracellular Ca^2+^. Average trace for *R/R_0_* values in SMCs treated with ET-1 (100 nM) and Tg (2 uM) in the absence of extracellular Ca^2+^. ET-1 induces a delayed bi-phasic drop in [Ca^2+^]_SR_, a small initial transient phase, followed by a large precipitous drop in [Ca^2+^]_SR_ (n = 6 independent experiments, mean ± SEM, *P*<0.005). Representative snapshots (FRET channel) of time-lapse movie (10 s intervals) illustrates an ET-1-induced regenerative Ca^2+^ depletion wave over time in a SMC (scale bars, 4.3 µm). At 300 s, the bathing solution is replaced by nominal Ca^2+^ free HEPES. At 600 s, ET-1 (100 nM) is added, which elicits a delayed decrease in luminal Ca^2+^ around 1160 s post-treatment. Immediately prior to the initiation of the depletion wave, a rapid transient increase in Ca^2+^ is observed at the point of origin (1200 s panel, circled area), followed by a significant drop due to wave development.

In order to distinguish the effects of ET-1 on luminal SR Ca^2+^ from those related to ET-1 stimulated Ca^2+^ entry from the extracellular space, we removed Ca^2+^ from the bathing solution, but without adding a chelator. Under these conditions, which inhibit SR Ca^2+^ refilling [18], ET-1 caused a large and delayed drop in [Ca^2+^]_SR_ (Fig. 4). The difference between ET-induced responses in the presence and absence of external Ca^2+^ is not surprising, because in the presence of external Ca^2+^, there is rapid Ca^2+^ cycling between the SR lumen and extracellular space [19], which appears to protect the SR from excessive Ca^2+^ depletion. In contrast, in nominally Ca^2+^ free condition, which inhibits SR refilling, ET-1 causes a much larger drop in [Ca^2+^]_SR_.

In about 80% of the cells, application of ET-1 after removal of external Ca^2+^ revealed a novel phenomenon that we refer to as the “SR Ca^2+^ depletion wave” (Fig. 4 and Movie S2). At 300 s, Ca^2+^ is removed from the bathing solution, and at 600 s, ET-1 (100 nM) is added. In the presence of ET-1, focal fluctuations in [Ca^2+^]_SR_ can be observed, and just before the onset of the Ca^2+^ depletion wave, [Ca^2+^]_SR_ is elevated at the site of wave initiation (Fig. 4, panel 1200 s). The SR depletion waves routinely originated close to the peri-nuclear region, rather than in the cell periphery.

**Figure 5:**
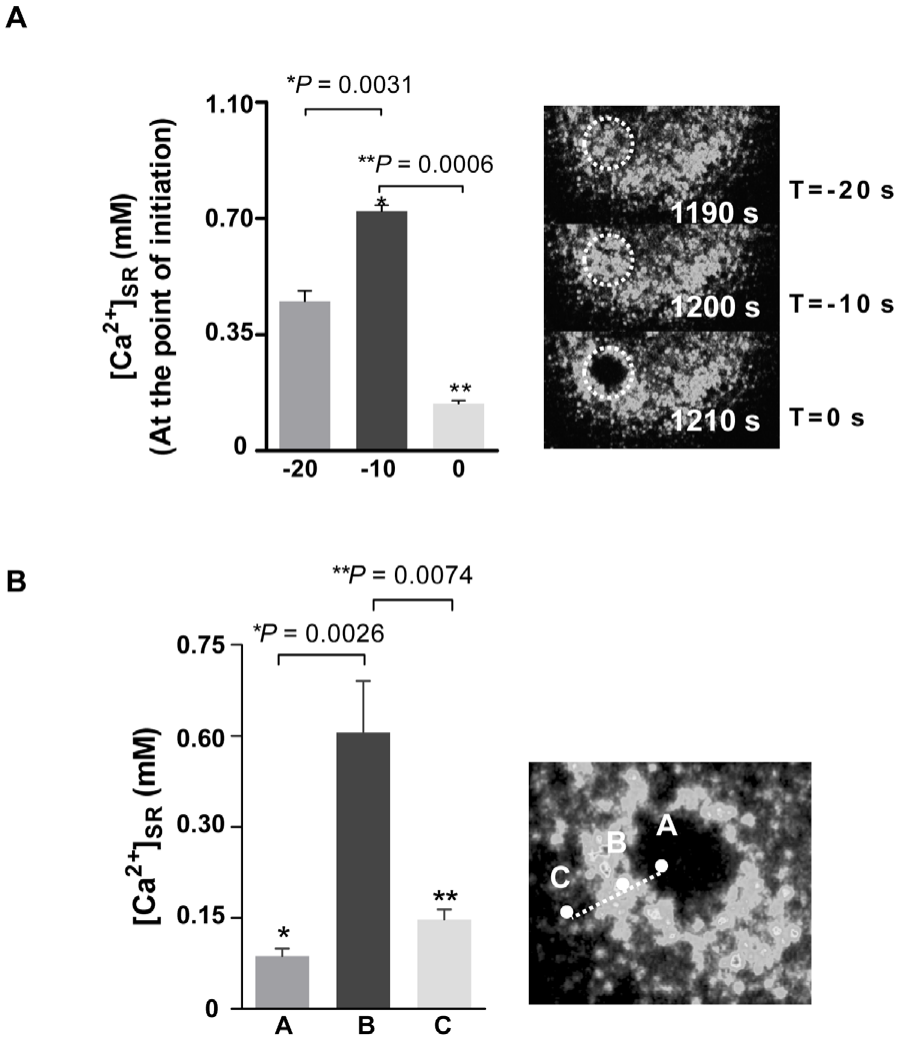
Characteristics of the ET-1-induced Ca^2+^ depletion waves. **A)** Average changes in Ca^2+^ signal intensity at the points of initiation. T = 0, T = −10 s, and T = −20 s refer to the time when the depletion wave first appears and 10 and 20 s prior to wave initiation, respectively. The Ca^2+^ signal significantly increases at T = −10 s, and drops to a low level as the waves initiate at T = 0 s (n = 5 cells from five independent experiments, mean ± SEM, *P = 0.0031, **P = 0.0006). **B)** *R/R_0_* values in three regions of the depletion wave (A: already depleted area; B: area that has taken up released Ca^2+^; C: area not yet affected by the wave B). At any given time point, the signal intensity at the rim (point B) is higher than both the already depleted area (point A) and the adjacent SR locus ahead of the wave (point C) (ROI = 4, n = 5 independent experiments, mean ± SEM, *P = 0.0026, **P = 0.0074).

**Figure 6:**
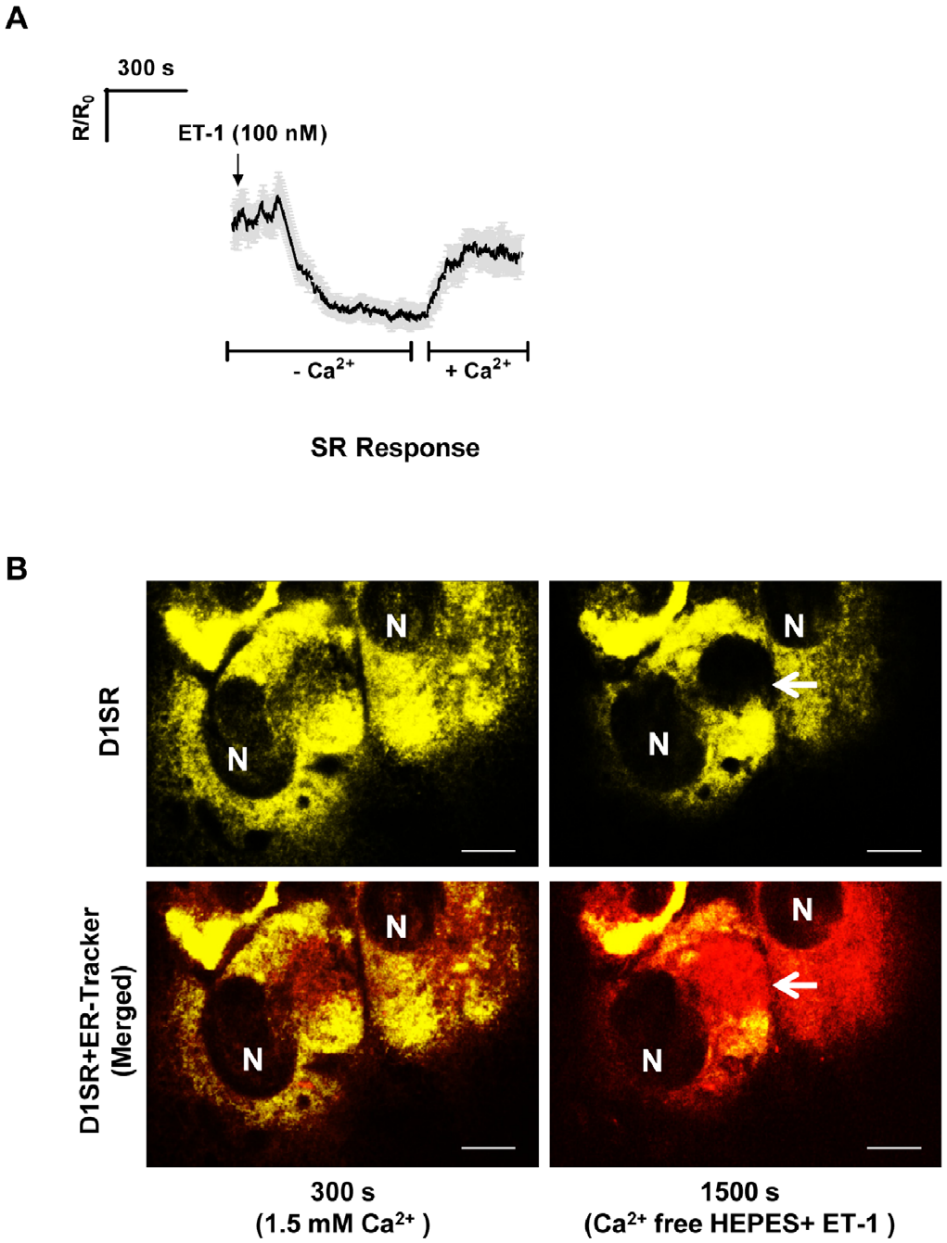
Refilling of SR Ca^2+^ and maintenance of SR structure following the depletion wave. **A)** The SR is refilled by subsequent re-perfusion of the cell culture with normal (1.5 mM) Ca^2+^ solution following depletion wave progression, confirming the reversibility of Ca^2+^ depletion wave. **B)** Live snapshots of overlay of D1SR and ER-Tracker signals in cultured SMCs showing that the SR luminal structure remains intact during and after the progression of ET-induced Ca^2+^ deletion wave (marked by white arrows) and in the absence of extracellular Ca^2+^. (scale bars, 15 µm).

Measurements of changes in the *R/R_0_* values at the point of wave initiation, confirmed a transient increase in [Ca^2+^]_SR_ to 700±100 µM immediately prior to wave initiation (Fig. 5A). Subsequently a depletion of [Ca^2+^]_SR_ occurs at the site of initiation (from 700±100 µM to 60±20 µM), from which a wave of Ca^2+^ depletion radiates out into the surrounding SR network (Fig. 4, panels 1210–1400 s). In the absence of external Ca^2+^ the extent of ET-1-induced [Ca^2+^]_SR_ depletion is similar to that induced by Tg. The depletion wave progresses in a roughly circular fashion and is always surrounded by a rim of elevated [Ca^2+^]_SR_. It thus appears that some of the Ca^2+^ released at the wave front is taken up by an adjacent, but not-yet-activated SR locus. Figure 5B shows that at any given time point of wave expansion, there are significant differences in [Ca^2+^]_SR_ between the depleted SR region (marked as A), the transiently refilled neighbouring SR locus (marked as B), and the next SR locus further ahead of the depletion wave (marked as C). After passage of the ET-induced Ca^2+^ depletion wave, the SR was capable of refilling upon subsequent re-perfusion with normal (1.5 mM) Ca^2+^ solution, confirming the reversibility of the observed SR depletion wave (Fig. 6A). Furthermore, the depletion waves recorded herein did not cause any long-lasting structural changes in SMCs, and the SR structure remained intact during the experiment (Fig. 6B).

### Ca^2+^ Depletion Waves in SMCs Propagate via IP_3_ Receptors

**Figure 7:**
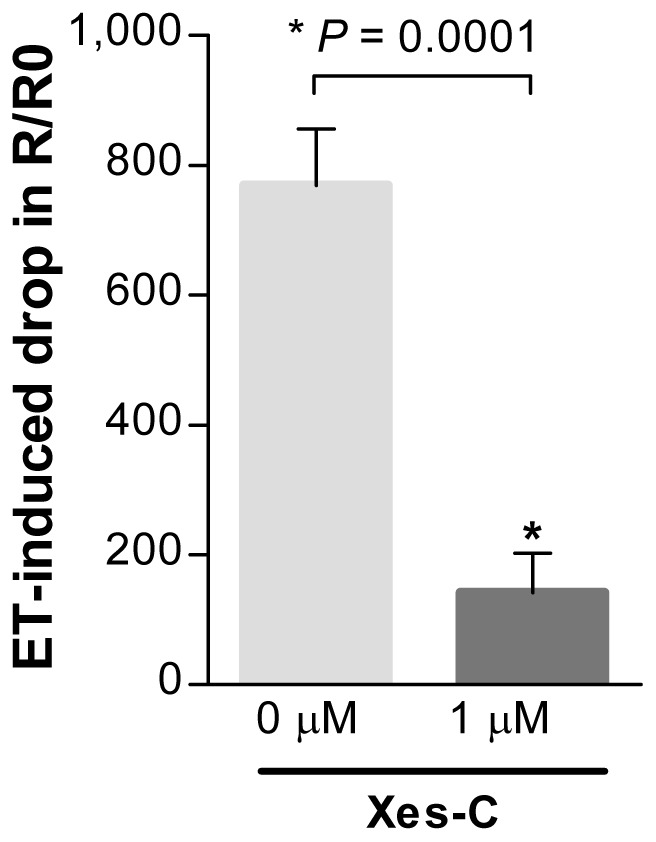
Ca^2+^ depletion waves propagate via IP_3_Rs. Inhibition of IP_3_Rs with Xes-C (1 µM) blocks ET-induced depletion waves as compared to vehicle-control groups. For Xes-C treated groups, cells were perfused with nominal Ca^2+^ free buffer containing 1 µM Xes-C starting 5 min prior to addition of ET-1 (n = 5 cells from five independent experiments, mean ± SEM, *P = 0.0001).

A plausible explanation for the initiation of SR Ca^2+^ depletion waves is that after the IP_3_ concentration builds up to a critical level and the cytoplasmic and SR Ca^2+^ concentrations at the initiation site reach a certain threshold values, the IP_3_Rs on the SR membrane open in a regenerative fashion; the wave is then propagated by CICR at IP_3_Rs. To test this hypothesis, we blocked the IP_3_Rs with a specific inhibitor xestospongin C (Xes-C) prior to the application of ET-1 (Fig. 7). Addition of Xes-C (1 µM) completely blocked ET-induced depletion waves, confirming that as for other VSM Ca^2+^ waves [8], [18], [20], the underlying mechanism is indeed mediated by CICR at IP_3_R. This notion was further corroborated by the observation that our cultured SMCs failed to respond to caffeine (data not shown), confirming the lack of functional RyRs in these cells, which is in agreement with a previous report by Vallot et al [21].

### Dynamics of the Ca^2+^ Depletion Waves in the SR Lumen

**Figure 8:**
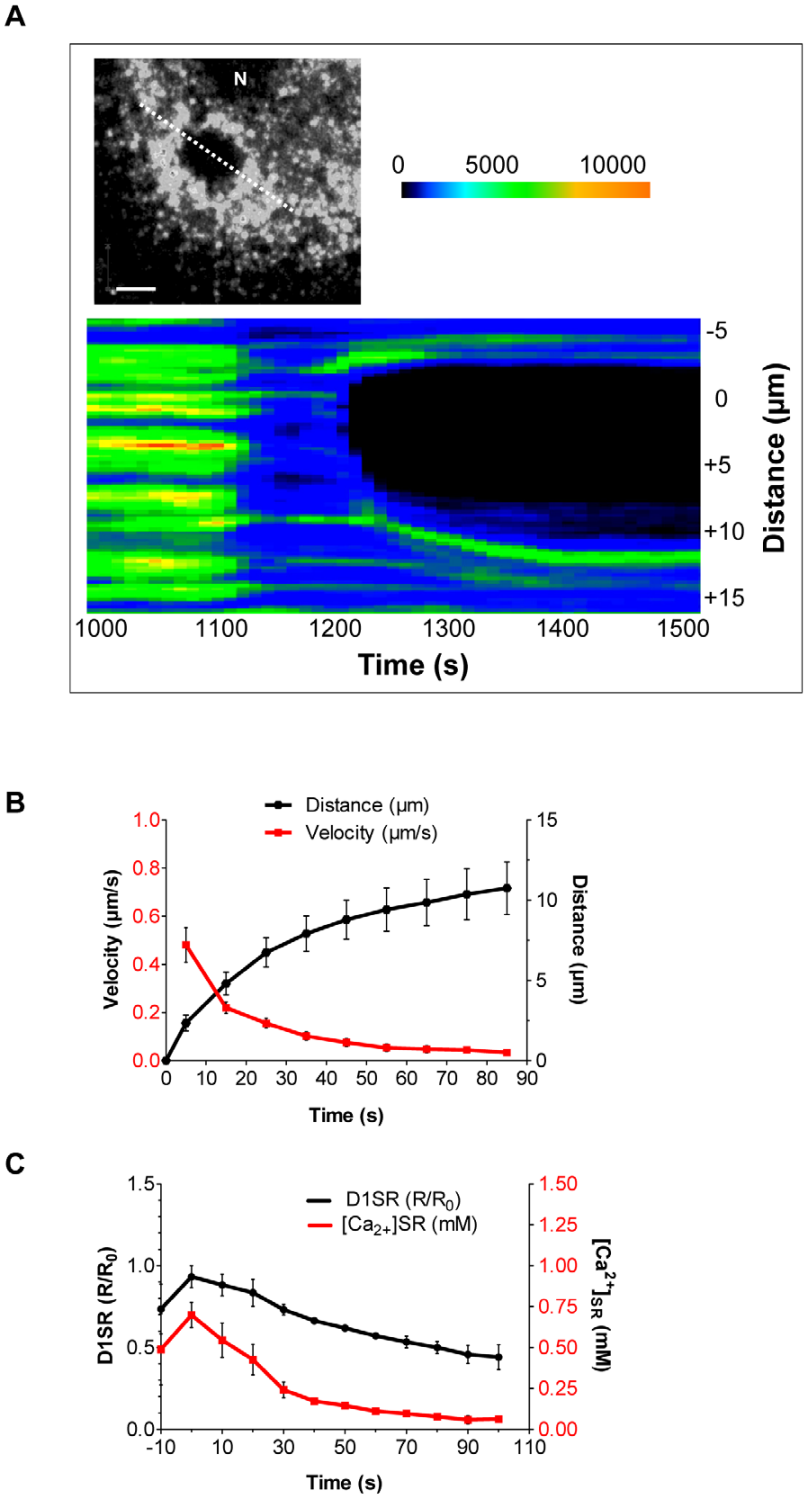
Dynamics of the Ca^2+^ depletion waves. **A)** Heat map generated from the time-lapse fluorescence microscopy movie (10 s intervals) of rat SMCs treated with ET-1 (100 nM) in nominally free extracellular Ca^2+^, showing that the SR depletion wave expands in a near circular fashion in both directions of the scan line (dotted white line). **B)** Average travel distance and velocity of ET-1-induced depletion wave *vs.* time. As the distance from the point of origin increases, the wave velocity decreases asymptotically to zero. **C)** Average values for *R/R_0_* and [Ca^2+^]_SR_ at the rim of the depletion wave also decreases as the wave expands over time (ROI = 5, n = 5 independent experiments, mean ± SEM, *P*<0.005).

Another instructive way of analyzing the [Ca^2+^]_SR_ depletion wave is to identify a line intersecting the site of wave initiation and recording the changes in *R/R_0_* values along this line *vs.* time (line scan). The resulting “heat map” illustrates that initially the area of [Ca^2+^]_SR_ depletion expands rapidly, but as time proceeds, it asymptotically approaches a final limit (Fig. 8A). Analysis of a number of such heat maps yields the average distance *vs.* time, as well as the velocity *vs.* time curves (Fig. 8B). Extrapolation of the velocity curve to the time of wave initiation (0 s), shows that the SR Ca^2+^ depletion wave has an initial velocity of about 0.7 µm/s, which falls below the range (2–30 µm/s) reported previously for intact smooth muscle Ca^2+^ waves in the presence of extracellular Ca^2+^ [22]. This could be explained by the fact that in our cultured VSMCs, ET-1 is a weak agonist, and a number of studies have shown that both the velocity and frequency of smooth muscle Ca^2+^ waves increase with the level of activation and the concentration of IP_3_ [8], [23]. The profound decrease in velocity over time is likely related to the decline of the [Ca^2+^]_SR_ at the rim as the wave progresses (Fig. 8C).

### Quantitative Model for Propagation of Ca^2+^ Depletion Waves

**Figure 9:**
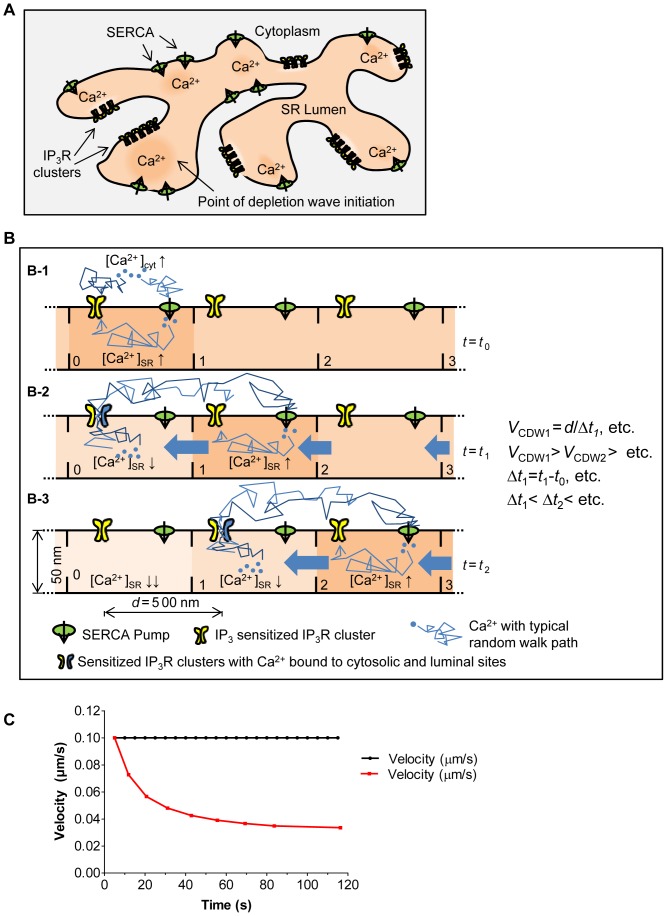
Simplified quantitative model for propagation of SR Ca^2+^ depletion waves. **A)** Schematic model of SR network depicting differential distribution of SR luminal Ca^2+^ sources (SERCA) and sinks (IP_3_Rs). **B)** We represent a linear section of SR as a series of compartments (labelled 0, 1, 2, etc), between which Ca^2+^ transport is allowed. Panels B-1, B-2, & B-3 represent the same portion of model SR at three different times, t_0_, t_1_ and t_2_. A wave initiating at compartment 0 in this model would move both left and right, but we focus only of the right-moving part for simplicity. Assuming that the [IP_3_]_cyt_ is such that the IP_3_R is already Ca^2+^-sensitized, a [Ca^2+^]_i_ fluctuation near the SR compartment 0 at *t* = *t*_0_ raises the [Ca^2+^]_SR_ from a resting level to a threshold level, and thereby, causes the observed transient rise (B-1). This rise is followed by depletion of Ca^2+^ from compartment 0. Partial equilibration of luminal Ca^2+^ then takes place (blue block arrows between compartments), accompanied by an increase in [Ca^2+^]_SR_ in compartment 1 (B-2). Subsequently, [Ca^2+^]_SR_ in compartment 1 reaches a threshold value leading to the releases of (some of) its Ca^2+^, which goes on to partially refill its nearest neighbouring locus (compartment 2), and so on (B-3). Ca^2+^ release from compartment 1 can trigger release from compartment 2, only after [Ca^2+^]_SR_ will have reached a critical level for Ca^2+^ release, which will take a time interval Δ*t*_2_ = t_2_−t_1_. Due to the Ca^2+^ passage from compartment 2 to 1, Δ*t*_2_ must be greater than *Δt_1_*, the interval it took to raise [Ca^2+^]_SR_ in 1 to the release level. Successive compartment refilling times to release level will get progressively longer because of the ability to re-equilibrate Ca^2+^ depletions. Assuming now that the CICR sustaining IP_3_R clusters are on average equidistant at a length *d* (500 nm) from one another, the Ca^2+^ depletion wave velocity (*v*_CDW_), as the wave reaches successive compartments, is given by *V*_CDW1_ *v*_CDW1_ = *d*/Δ*t_1_*> *v*_CDW2_> …>*etc*. Under the conditions of the reported experiments, disallowing Ca^2+^ communication between SR compartments in this model would translate in an equal interval Δ*t* between the wave arrival at each compartment, and therefore in a constant wave velocity (*v*_CDW_ = *d*/Δ*t*). **C)** Ca^2+^ depletion wave velocity *vs.* time calculated from our model, considering Ca^2+^ communicating (solid curve) or Ca^2+^ tight (dashed) SR compartments.

The observed transient increase in [Ca^2+^]_SR_ at both the origin and the rim of the depletion wave (Fig. 5A and 5B) suggests an important role for local [Ca^2+^]_SR_ elevation during initiation and propagation of regenerative IP_3_R-mediated Ca^2+^ release. Since in cytoplasmic Ca^2+^ oscillations, the latency period is related to the inter-spike interval [24], which is, in turn, regulated by the SR Ca^2+^ content [24], the VSMCs activity may well be controlled by focal fluctuations in [Ca^2+^]_SR_. In line with previous reports [25], [26], we propose that these fluctuations are generated in part by a differential distribution of SR luminal Ca^2+^ sinks (clusters of IP_3_Rs), and Ca^2+^ sources (SERCA) (Fig. 9A). Stochastic opening of individual IP_3_Rs (which yield cytoplasmic Ca^2+^ blips) [25], [26] would add variability to focal [Ca^2+^]_SR_. Whenever an increase in local [Ca^2+^]_SR_ exceeds the threshold for regenerative opening of an IP_3_R cluster, sufficient Ca^2+^ would be released to initiate a regenerative Ca^2+^ wave.

Generation of a wave according to this mechanism, and possessing the features of deceleration and decreasing intensity at the progressing rim as we observe, implies a continuity of the SR lumen. Since under control conditions the non-activated SR remains loaded with Ca^2+^ because of continual activity of SERCA, the observed decline in [Ca^2+^]_SR_ at the rim of the depletion wave (Fig. 8C) is a strong indication that a re-equilibration of the SR Ca^2+^ content is taking place within the SR lumen. Only in this manner, when removal of extracellular Ca^2+^ blocks SR refilling, then opening of IP_3_Rs at the wave front would partially deplete the SR located just ahead of the wave.

To analyze this proposed mechanism, we developed a preliminary and simplified two-dimensional quantitative model for the propagation of the observed depletion waves, which is based on previous observations and suggested models arguing for 1) a continuous SR lumen in regards to Ca^2+^ transport, 2) the appearance of functionally segregated compartments in the SR of SMCs, and 3) the existence of a luminal Ca^2+^ binding site of the IP_3_R [27], [28], [29]. The essential steps of this model are presented in Figure 9B, and the generated trace for the velocity of the depletion wave is presented in Figure 9C.

Using the symbols and data in Table 1, our model assumes that, before any Ca^2+^ depletion wave (CDW) event, the [Ca^2+^]_SR_ is at a normal resting level (C_SR_) and that it needs to reach a critical level (C^*^_SR_) for release. Then, for example, following the depletion of a given SR compartment, for a nearest neighbouring compartment to release, its [Ca^2+^]_SR_ needs to change by C^*^_SR_−C_SR_ = 200 µM, which translates to about 1500 Ca^2+^. For the sake of order-of-magnitude calculations, let us say that SERCA pumps operating at 300 s^−1^ would take a time interval Δ*t_1_* = 5 s to cause a 200 µM [Ca^2+^]_SR_ change. We calculate the depletion wave velocity, *v*_CDW_, as the inter-IP_3_R-cluster distance, *d*, divided by the time, Δ*t*, taken to raise the [Ca^2+^]_SR_ in the new compartment from normal to critical: *v*_CDW_ = *d/*Δ*t*. For the sake of completeness, the wave velocity should also include a component for the Ca^2+^ diffusion time from cluster to cluster. This component is however of the order of milliseconds and therefore negligible when compared to SR compartment refill times.

**Table 1:** Symbols and data for the quantitative Model.

Quantity	Data	References
SR lumen cross-section	50 nm×50 nm	[46], [47]
IP_3_R cluster-cluster distance, *d*	500 nm	[46]
SR compartment volume	50 nm×50 nm×500 nm	[46], [47], [48]
SERCA refill rate	300 s^−1^	[46]
Normal [Ca^2+^]_SR_, C_SR_	500 µM	our observation
Critical [Ca^2+^]_SR,_ C*_SR_	700 µM	our observation
Ca^2+^ shift from 1 to 0(see Fig. 9B)	20%	our choice
Ca^2+^ shift from 2	5%	our choice
Ca^2+^ shift from 3	1%	our choice

In this manner, for one depletion wave “step” in our model (from compartment 0 to 1 in Figure 9B), *v*_CDW,1_ = *d/*Δ*t_1_* = 0.1 µm/s. If we now assume that SR compartments 2, 3, etc lose part of their Ca^2+^ toward the depleted neighboring SR compartments by the percentages indicated in Table 1 (these values are arbitrarily chosen), their “normal” [Ca^2+^]_SR_ will be C_2,SR_, C_3,SR_, etc., where these values are *lower* than C_SR_. The same line of reasoning then suggests that, for compartment 2 to release, its [Ca^2+^]_SR_ needs to change by C^*^_SR_− C_2,SR_>200 µM, which will take a time Δ*t_2_*>Δ*t_1_* and, in turn, yields a velocity for the second step as *v*_CDW,2_ = *d/*Δ*t_2_*<*v*_CDW,1_. To consider what would happen in a portion of SR with Ca^2+^ tight compartments, to a first degree of approximation, we can re-use the model above with 0% Ca^2+^ communication between compartments. It should be evident then that the interval needed to refill each compartment before release is the same as the putative wave progresses. Since the average inter-cluster distance is the same, the resulting wave velocity would be a constant value v_CDW_ = *d*/Δ*t* at each step. The results reported in Figure 9C were obtained by following this step-by-step procedure for about 100 seconds, which confirms that introduction of such luminal Ca^2+^ flux into the model indeed caused incremental decrease of the simulated Ca^2+^ depletion wave velocity (Fig. 9C, red trace).

**Figure 10:**
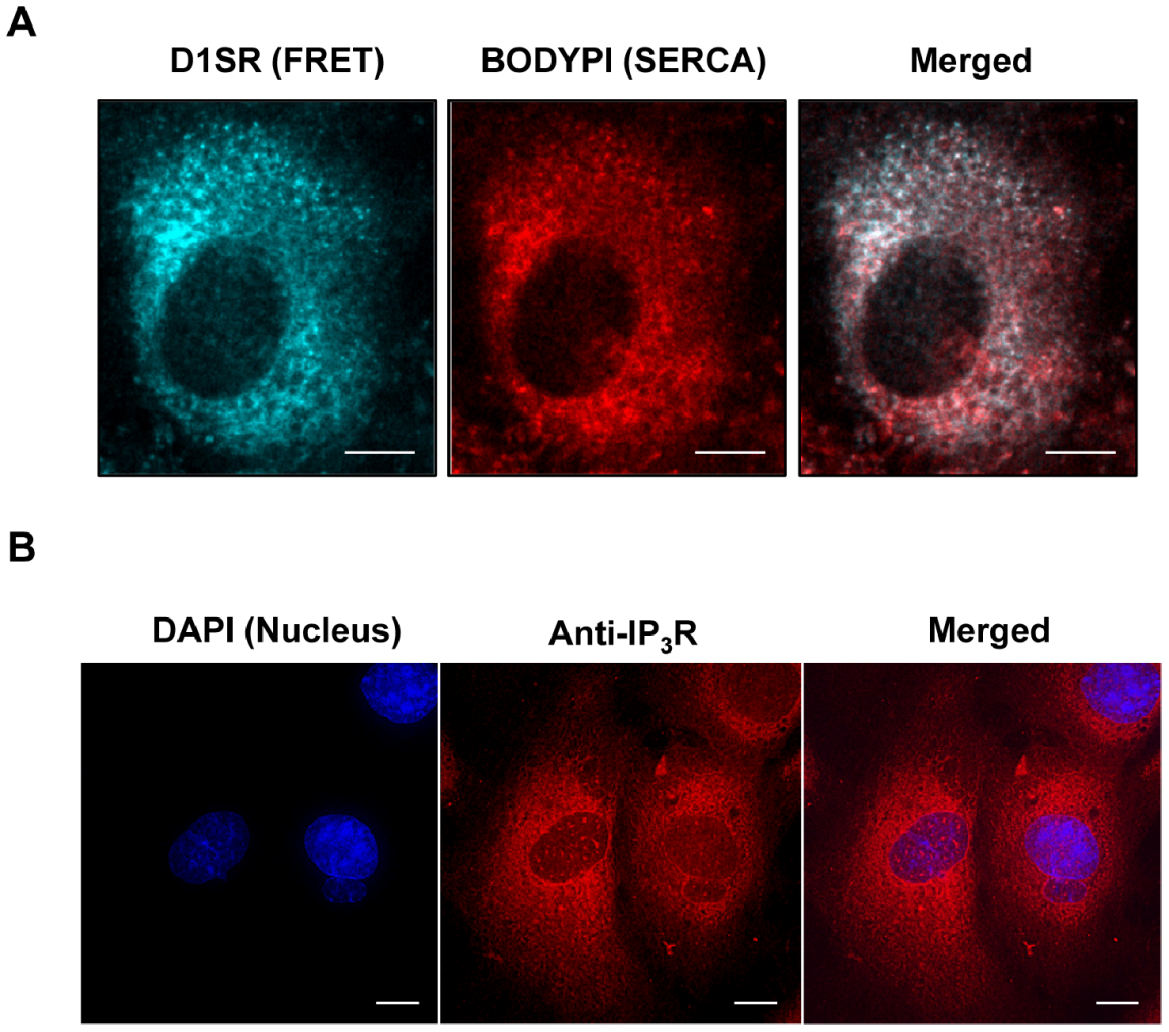
Distribution of SERCA and IP_3_Rs in cultured rat aortic SMCs. **A)** Live snapshots of cultured rat aortic SMCs transfected with D1SR construct and treated with 1 µM of BODIPY® TR-X thapsigargin showing the distribution of SERCA in cultured SMCs. SERCA distribution follows the same pattern as SR luminal network as indicated by expression of D1SR indicator (FRET channel) within the SR lumen (scale bars, 10 µm). **B)** Receptors for IP_3_ (IP_3_Rs) were immuno-labelled using rabbit polyclonal antibody against IP_3_R type I and Alexa-594 secondary antibody (Red). Images are representative of 29 cells imaged from four independent SMC cultures, and are shown as maximal intensity projections of de-convolved image stacks. Nucleus is labeled using DAPI (blue) (scale bars, 5 µm).

Our proposed model clearly depends on populations of IP_3_Rs and SERCA spread over the entire SR network. This appears to be the case for the SMCs used in this study as both SERCA (Fig. 10A) and IP_3_Rs (Fig. 10B) display the same diffuse distribution as the D1SR and ER-Tracker.

### Arrest of the ET-induced SR Depletion Wave at the Border of the Nuclear Envelope

**Figure 11:**
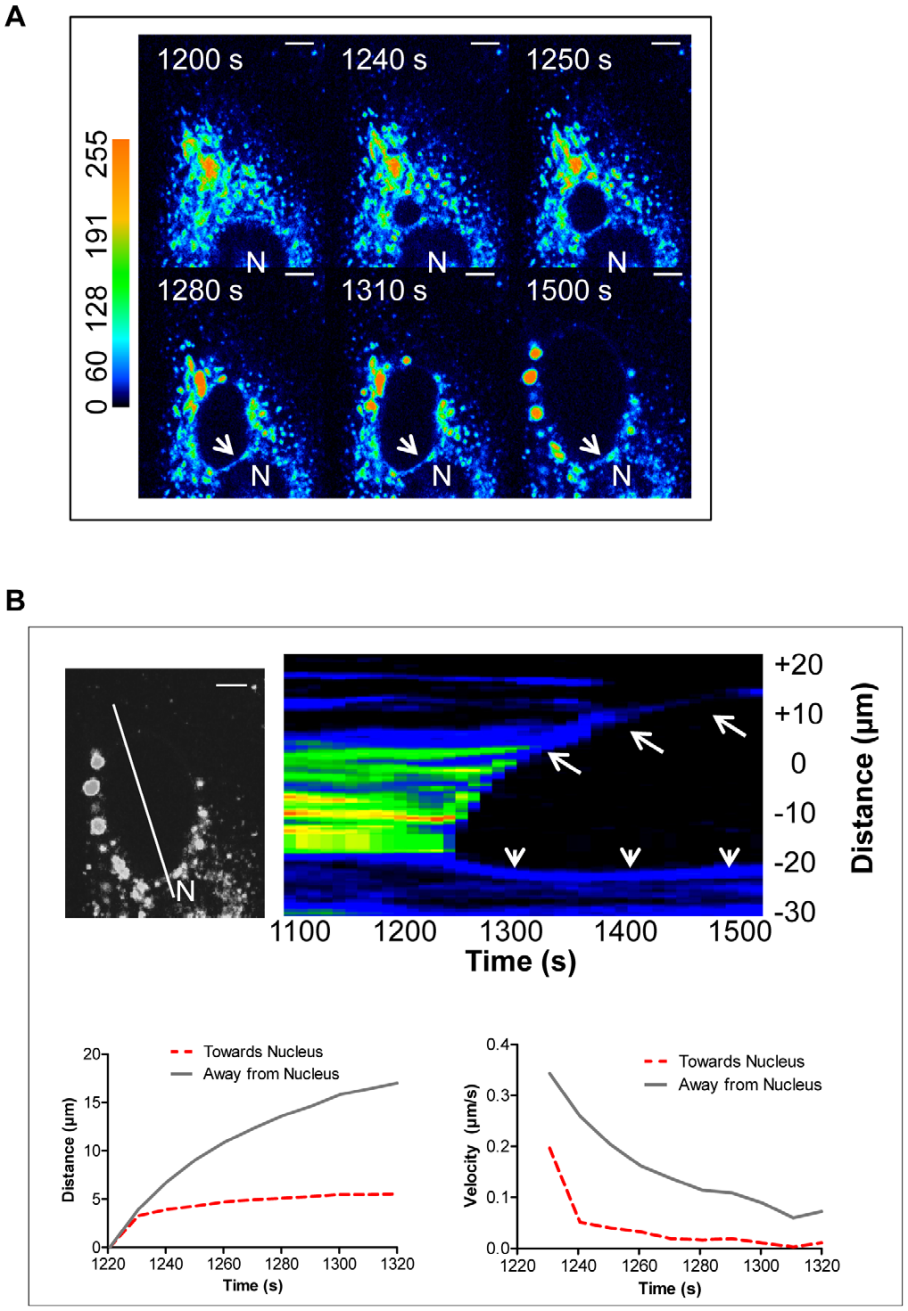
Arrest of the *Ca^2+^* depletion waves at the border of the nuclear envelope. **A)** Snapshots (FRET channel) of SMCs treated with ET-1 (100 nM) in the absence of extracellular Ca^2+^. The depletion wave is arrested at the nuclear envelope (white arrows) but progresses in the opposite upward direction (scale bars, 5 µm; N, nucleus). **B)** Line scan of Ca^2+^ signal through the point of origin of the depletion wave and heat map of the arrested wave. The depletion wave is unable to proceed through nuclear region and arrested at the nuclear border creating an almost straight line of Ca^2+^-rich areas (white arrowheads), while progressing in the opposite direction (white arrows) (scale bars, 5 µm). Traces compare the distance travelled and velocity of the wave over time toward and away from the nuclear envelope from the point of origin.

**Figure 12:**
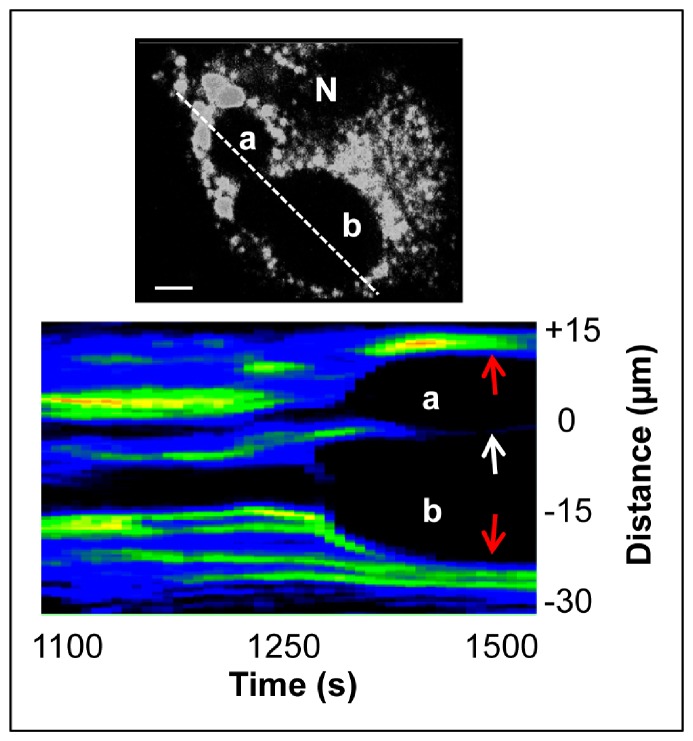
Colliding of neighbouring Ca^2+^ depletion waves. Line scan of Ca^2+^ signal through the point of origin of two neighbouring depletion waves (a & b) and the heat map of the colliding waves accentuate that the bright rim between two neighbouring waves disappears over time (white arrow), while the rims facing the plasma membrane persist (red arrows) (N, nucleus, scale bars, 5 µm).

Close inspection of the SR depletion waves also provides novel insight into sub-cellular differential Ca^2+^ signalling. Figure 11A presents snapshots of a depletion wave, which originates very close to the nucleus (see Movie S3). In this particular case, the shape of the wave front becomes oblong instead of circular, as it fails to involve the nuclear envelope (white arrows), and proceeds in the opposite peripheral direction. The asymmetry of this particular wave is clearly demonstrated by the heat map in Figure 11B. As shown, the distance traveled by the wave and its velocity drop dramatically when the wave collides with the nuclear envelope, without depleting its luminal Ca^2+^ content. In contrast, the pattern of two neighbouring SR depletion waves colliding with each other is clearly different from the case when a depletion wave encounters the nuclear envelope. As shown in Figure 12, in some cells, multiple Ca^2+^ depletion waves develop independently. In these cells, a line scan of the Ca^2+^ signal through the point of origin and through the border between neighbouring waves (a & b) shows that the depleted areas of colliding waves coalesce (white arrow) without a dividing line of Ca^2+^ loaded SR as seen at the periphery of both depletion waves (red arrows).

## Discussion

In this first report of Ca^2+^ depletion waves in the smooth muscle sarcoplasmic reticulum, we describe a crucial role for luminal [Ca^2+^]_SR_ in the initiation and propagation of cytoplasmic Ca^2+^ waves. This additional insight was gained because the dynamics of SR Ca^2+^ depletion do not simply mirror [Ca^2+^]_i_ elevations, but display their own unique characteristics. The disparity between the [Ca^2+^]_i_ and [Ca^2+^]_SR_ transients is most likely due to significant contributions by plasma membrane Ca^2+^ fluxes to smooth muscle Ca^2+^ homeostasis and excitation.

Iino’s hypothesis that Ca^2+^ waves in VSM are propagated by regenerative CICR at the IP_3_Rs [4] was recently corroborated by a well-controlled study by McCarron and collaborators on freshly isolated SMCs, voltage clamped and incubated in a Ca^2+^ free medium [22]. Progression of the wave front by CICR requires that the released Ca^2+^ raises the local [Ca^2+^]_i_ in the neighbouring nano-domain occupied by IP_3_Rs, which are about to be activated. Although such spatio-temporal resolution of cytoplasmic Ca^2+^ waves has not yet been reported, our data conclusively show that this is indeed the case. The only possible explanation for the increased [Ca^2+^]_SR_ at the wave rim is that the SERCA pumps respond to the local [Ca^2+^]_i_ elevations before activation of IP_3_R ahead of the Ca^2+^ wave. Our observation of a diffuse distribution of both SERCA and IP_3_R over the entire SR (see Fig. 10) corroborates this view. Moreover, our conclusions show Ca^2+^ diffuses from an elevated [Ca^2+^]_i_ site, to a neighbouring site containing an IP_3_R cluster more rapidly than the propagation of a depletion wave. This supports the mechanism of a wave of CICR, and is experimentally confirmed by the increased [Ca^2+^]_SR_ at the wave rim due to SERCA-mediated Ca^2+^ uptake in response to the local [Ca^2+^]_i_ elevation before activation of IP_3_Rs, ahead of the Ca^2+^ wave.

The transient elevations of [Ca^2+^]_SR_ at the site of wave initiation and at the wave front, directly demonstrate local interactions between adjacent SR sites, which depend on SR luminal continuity and in principle could be readily influenced by other Ca^2+^ transporting organelles, such as mitochondria and lysosomes in shaping the smooth muscle Ca^2+^ signal [30], [31], [32], [33], [34]. It is evident that the elevated [Ca^2+^]_SR_ at the origin and at the rim of the wave plays a crucial role in wave initiation and propagation, underscoring the importance of previous reports demonstrating that [Ca^2+^]_SR_ has a stimulatory role in IP_3_R-mediated Ca^2+^ release [35], [36], [37].

In fact, several reports make a strong case for the existence of a luminal excitatory Ca^2+^-binding site on IP_3_R, which would provide a straightforward explanation for the stimulating effects of [Ca^2+^]_SR_ [27], [28], [38], [39]. Regulation of IP_3_R by luminal Ca^2+^ suggests a mechanism for Ca^2+^ wave initiation based on local [Ca^2+^]_SR_ fluctuations caused by random opening of IP_3_Rs and separation of Ca^2+^ sources (SERCA) and sinks (IP_3_Rs) in the walls of the SR lumen (see Fig. 9). It is conceivable that the “Frequent Discharge Sites” observed by Bolton and coworkers [40] were due to specific local ultra-structural characteristics of the SR which favour focal [Ca^2+^]_SR_ fluctuations. One of these is closeness to the nucleus, which has been shown in HeLa cells to delay the decay of IP_3_R-generated Ca^2+^ puffs, due to decreased sequestering/buffering [25], [26].

A relevant question is why these waves were observed only in the absence of external Ca^2+^. One plausible answer is that removal of external Ca^2+^ causes marked lowering of [Ca^2+^]_i_ and thereby delays SR Ca^2+^ release stimulated by ET-1. In our VSMC preparation, we have shown that the release is mediated by opening of IP_3_Rs (see Fig. 7). It is likely that the reduced [Ca^2+^]_i_ due to removal of external Ca^2+^ lowers the rate of IP_3_ synthesis by phospholypase C, and thus increases the time required for IP_3_ concentration to reach a threshold value [41], [42]. The latency period would be further increased because the low [Ca^2+^]_i_ would also raise the threshold for IP_3_-mediated activation. The consequently slower rates of spontaneous discharge combined with the lack of SR refilling would greatly enhance the development of Ca^2+^ depletion waves. On the other hand, in the presence of extracellular Ca^2+^, rapid SR refilling could obscure the depletion process. In addition, activation of clusters of IP_3_Rs at much higher frequency would prevent the development of full Ca^2+^ depletion waves. Since it was essential to separate trans-plasma membrane fluxes from the Ca^2+^ release process, we simply removed the extra-cellular Ca^2+^ component, a procedure which we and numerous previous studies have shown to not have deleterious effects on the SR [16], [20], [43], [44], [45]. Thus, we conclude that the observed Ca^2+^ depletion waves provide valuable new insight into a regulatory role of [Ca^2+^]_SR_ in the processes of Ca^2+^ wave initiation and propagation.

Finally, the arrest of the SR depletion wave at the border of the nuclear envelope indicates that, as has been shown previously [17], the two membrane systems may differ with respect to their respective Ca^2+^ transport mechanisms. Figure 10B strongly suggests that the nuclear envelope does contain IP_3_R, but whether these are located in the membrane facing the cytoplasm or the inner membrane facing the nucleoplasm is an open question. Nevertheless, the observation that the wave does not deplete the nuclear envelope, could be of physiological importance in terms of segregation of dynamic Ca^2+^ signalling of vasoconstriction from functions such as gene transcription.

In conclusion, the findings presented in this study provide new insight into the mechanisms whereby the ER/SR, the main Ca^2+^ regulatory organelle in living cells, determines spatial and temporal characteristics of cellular Ca^2+^ signalling, which differentially control cellular migration, growth, proliferation and apoptosis in health and disease.

## Supporting Information

Movie S1 **3-D structure of rat SMCs transfected with D1SR.**
(WMV)

Movie S2 **ET-induced SR calcium depletion wave.**
(WMV)

Movie S3 **Arrest of depletion wave at the nuclear envelope.**
(WMV)
